# Baclofen Response in Alcohol Dependent Patients Concurrently Receiving Antidepressants: Secondary Analysis From the BacALD Study

**DOI:** 10.3389/fpsyt.2018.00576

**Published:** 2018-11-19

**Authors:** Sovandara Heng, Nazila Jamshidi, Andrew Baillie, Eva Louie, Glenys Dore, Nghi Phung, Paul S. Haber, Kirsten C. Morley

**Affiliations:** ^1^NHMRC Centre of Research Excellence in Mental Health and Substance Use, Central Clinical School, Sydney Medical School, University of Sydney, Sydney, NSW, Australia; ^2^Drug Health Services, Royal Prince Alfred Hospital, Sydney Local Health District, Sydney, NSW, Australia; ^3^Faculty of Health Sciences, NHMRC Centre of Research Excellence in Mental Health and Substance Use, University of Sydney, Sydney, NSW, Australia; ^4^Herbert Street Clinic, Royal North Shore Hospital, Sydney, NSW, Australia; ^5^Centre for Addiction Medicine, Westmead Hospital, Sydney, NSW, Australia

**Keywords:** baclofen, alcohol dependence, antidepressants, depression, anxiety, comorbidity, treatment

## Abstract

**Background and Aims:** There is little information with regards to the efficacy of baclofen among alcohol patients concurrently receiving antidepressants (AD). The present study aimed to conduct a secondary analysis of the moderating role of antidepressants in the BacALD trial which evaluated the efficacy of baclofen to reduce alcohol consumption in alcohol dependent patients.

**Methods:** Alcohol dependent patients (*N* = 104) were treated for 12 weeks with 30 mg/day of baclofen (21 = AD and 15 = no AD), 75 mg baclofen (19 = AD and 16 = no AD) or placebo (17 = AD and 16 = no AD). Patients were included in the trial if they were concurrently receiving anti-depressants upon enrolment but were excluded if they commenced antidepressants 2 months prior to enrolment. Patients were also excluded in the case of concurrent psychotropic medications, active major mental disorder such as bipolar disorder, psychosis, or history of suicide attempt. Predefined primary outcomes included time to lapse (any drinking), relapse (>5 drinks per day in men and >4 in women). Other outcomes included drinks per drinking day, number of heavy drinking days, and percentage days abstinent and frequency of adverse events.

**Results:** For the number of days to first lapse, there was a trend of significance for the interaction baclofen × AD (Log Rank: χ^2^ = 2.98, *P* = 0.08, OR: 0.41, 95%CI: 0.15–1.12). For the number of days to relapse, there was a trend of significance for the interaction of baclofen × AD (Log Rank: χ^2^ = 3.72, *P* = 0.05, OR: 3.40, 95%CI: 1.01–11.46). Placing significant baseline variables into the models as covariates (tobacco, ALD) weakened these interactions (*P*'s > 0.15). There were no significant effects of ADs on the frequency of adverse events reported (*P*'s > 0.19).

**Conclusion:** Concurrent receipt of ADs commenced more than 2 months prior to baclofen treatment did not negatively impact on drinking outcomes. Future research examining the interaction between commencing ADs during baclofen treatment on alcohol dependent patients is required.

**Trial Registration:** ClinicalTrials.gov, NCT01711125, https://clinicaltrials.gov/ct2/show/NCT01711125

## Introduction

World-wide, harmful use of alcohol is responsible for 5.9% of all deaths and is a causal factor in a larger number of disease and injury conditions (1). Alcohol dependence is a common disorder characterized most often by chronic relapses to heavy alcohol consumption (2). Comorbidity of alcohol use disorders (AUDs) and mental illness such as major depression are highly prevalent in treatment settings (3, 4) and these individuals present with greater symptom complexity and poorer outcomes (3, 5).

Pharmacological treatment options for these complex patients are limited and little is known about the effectiveness of current pharmacological approaches for alcohol problems. Many of the randomized controlled trials exclude patients that are concurrently receiving antidepressants and/or with comorbid major depression despite the fact that a substantial percentage of treatment-seeking AUD patients fall into these categories. In our previous clinical trials, up to 50% of alcohol dependent patients were receiving anti-depressants prior to treatment seeking for alcohol problems (6–9).

Baclofen, a selective GABA_B_ receptor agonist, has emerged as a potential treatment for alcohol dependence (10). There has been expanded utilization including increased use in primary care (11). Nonetheless, there has been several reports of adverse events in AUD patients with psychiatric comorbidity including mood disorders (12) and also a higher rate of self-poising in those with concomitant borderline personality disorder and AUD (13). Moreover, a recent report from the Australian Poisons Information Centre (PIC) observed that antidepressants represented 17% of co-ingestants associated with baclofen toxicity (14). Thus, although treatment with baclofen appears to be popular in the community, there remain several important clinical issues in need of exploration. Improvement in the ability to predict baclofen response particularly among those with common comorbid mood conditions or concurrent receipt of antidepressants would be of clinical importance. No clinical trials have examined the role of antidepressants during treatment of alcohol dependence with baclofen.

The current study thus aimed to retrospectively examine the moderating role of concurrent antidepressant use on baclofen treatment response. We conducted a secondary analysis of the Baclofen in the treatment of Alcohol Liver Disease (BacALD) randomized controlled trial (15) which demonstrated a beneficial effect of baclofen on treatment outcomes (6).

## Methods

### Design

**Figure 1:**
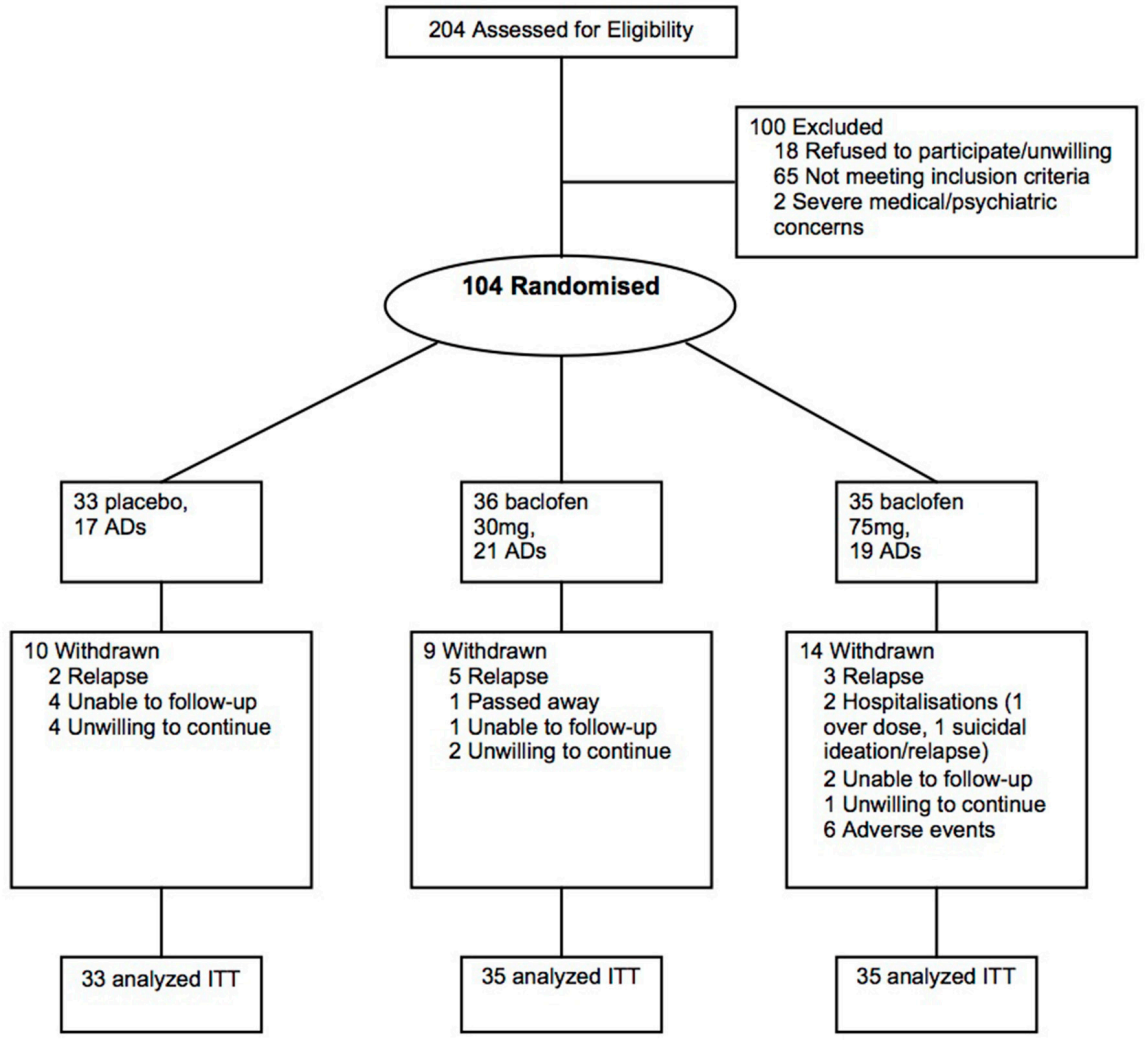
Flow of participants through the randomized controlled 12-week trial of placebo, baclofen 30, and baclofen 75 in the treatment of alcohol dependence, including receipt of concurrent antidepressants (AD) at least two months prior to trial commencement.

The main study rationale, design, and methods have been previously detailed (15) and the primary outcomes reported (6). In brief, after baseline assessment, eligible alcohol-dependent individuals were randomized to placebo, baclofen 30 mg (10 t.i.d.) and baclofen 75 mg (25 t.i.d.) for 12 weeks. Of the 104 individuals randomized in the main trial, 57 individuals were receiving concurrent antidepressants (AD) prior to randomization (Figure 1). The list of antidepressants prescribed is depicted in Table 1. No individuals commenced AD while on the trial. The study was approved by the Human Ethics Review Committee of the Sydney Local Health District, Northern Sydney Local Health District and South Western Sydney Local Health District (X11-0154 & X07-0041 & X01-0262) and the main trial was registered in the Clinical Trials Registry (NCT01711125). The study involved off-label use of a registered medication in Australia and approval was given under the Clinical Trial Notification (CTN) scheme of the Therapeutics Goods Administration (TGA) (2013/0060).

**Table 1:** List of antidepressants participants were receiving at least two months prior to trial commencement.

**Compound**	**Antidepressant class**
Allegron (nortriptyline)	TCA
Arapax (paroxetine)	SSRI
Brintellix (vortioxetine)	SSRI
Cipramil (citalopram)	SSRI
Effexor (venlafaxine)	SNRI
Elavil (amitrptyline)	TCA
Lexapro (escitalopram)	SSRI
Pristiq (desvenlafaxine)	SNRI
Prozac (fluoxetine)	SSRI
Remeron (mirtazapine)	TCA
Zoloft (sertraline)	SSRI

*TCA, Tricyclic antidepressant; SSRI, Selective Serotonin Reuptake Inhibitor; SNRI, Serotonin and norepinephrine reuptake inhibitor*.

### Participants and procedure

Participants were Australian Caucasian men and women who had attended an inpatient detoxification program, outpatient treatment or follow-up or who had responded to advertising. All participants signed informed consents.

Inclusion criteria: (i) Alcohol dependence according to the ICD-10 criteria; (ii) Age 18–75; (iii) Adequate cognition and English language skills to give valid consent and complete research interviews; (iv) Willingness to give written informed consent; (v) Abstinence from alcohol for between 3 and 21 days; (vi) Resolution of any clinically evident alcohol withdrawal (CIWA-AR); (vii) Not < 48 h after ceasing any diazepam required for withdrawal management. For stratification, alcoholic liver disease (ALD) was defined as the presence of symptoms and/or signs referable to liver disease or its complications with or without cirrhosis. Alcohol use was considered to play a major etiological role and exceeded an average of 60 g/day in women and 80 g/day in men for >10 years. If other co-factors such as chronic hepatitis C were present, a significant contribution of alcohol to liver disease was considered present if a period of supervised abstinence (e.g., in hospital) led to a ≥50% improvement in liver enzymes. Exclusion criteria: (i) Active major mental disorder associated with psychosis or significant suicide risk, (ii) Pregnancy or lactation, (iii) Concurrent use of any psychotropic medication other than antidepressants (provided these are taken at stable doses for at least 2 months); (iv) Unstable substance use; (v) Clinical evidence of persisting hepatic encephalopathy (drowsiness, sleep inversion or asterixis); (vi) Pending incarceration; (vii) Lack of stable housing, (viii) Peptic ulcer; (ix) Unstable diabetes mellitus.

### Assessments

A detailed list of assessments has been outlined previously (15). Briefly, the outcomes for this study were derived from drinking measures in the Time Line Follow Back [TLFB] (16) obtained from structured interviews at baseline and during the 12-week trial period (weeks 1, 3, 6, 9, 12). Depression as measured by the Depression Anxiety Stress Scale (DASS (17). In addition, trained interviewers conducted a structured psychiatric diagnostic interview using the Mini International Neuropsychiatric Interview (M.I.N.I.) (18). Compliance was assessed by self-report, pill count of the returned medication package, the daily monitoring diary and urinary analysis of baclofen levels in a randomly selected 50% of participants. Researchers, clinicians, and participants were blinded from treatment allocation.

### Interventions

Participants were allocated 1:1:1 as per a computer-generated randomization sequence provided to the hospital clinical trials pharmacist. Participants in the baclofen 30 mg/day or 75 mg group took a capsule of 10 or 25 mg, respectively, 1 × day for the first 2 days, 2 × day on days 3–4, 3 × day on days 5–80, 2 × day on days 81–82 and finally 1 × day for the last 2 days. The placebo pills, which were identical in appearance, were also titrated upward and downward to maintain the double blind. All participants received medical care typically available at hospital based drug and alcohol treatment services in Sydney, Australia. All participants received 1 medical assessment and 5 follow-up medical reviews over the 12 week treatment period, held at weeks 1, 3, 6, 9, 12. Participants were medically monitored for adverse events and prescribed the study medication at each appointment. Participants who experienced moderate side effects had their dose reduced according to physician judgment. All participants received brief compliance therapy, a 4–6 session intervention lasting 20–60 min focused on enhancing medication compliance (such as targeting ambivalence and misperceptions about medication). Participants were encouraged to defer concurrent psychotherapy until at least week 6 of the trial.

### Outcomes measures

As per the main study published results, the primary outcomes include time to first lapse (1 drink); time to relapse (>4 drinks for women, >5 drinks for men); average drinks per drinking day (at wk 12 follow-up) and number of heavy drinking days (at wk 12 follow-up); percentage days abstinent (over the wk 12 trial).

### Statistical analysis

Analyses were performed on an intention-to-treat basis including all participants who took at least one dose of medication. As previously outlined and published in the main trial results (6, 15), the analyses of primary outcomes included placebo vs. baclofen (composite of the two doses). Analysis of variance (ANOVA) for continuous characteristics and χ^2^-tests for categorical variables were conducted to determine differences between groups at baseline. Cox regression, which allows for the analysis of the effect of several risk factors on survival, was conducted to examine the effect of baclofen (PL vs. BAC) × AD on length of time to relapse and length of time to lapse (calculated from day 1 to day 84 on the trial). Participants were censored if they did not experience the outcome (relapse or lapse) on or before day 84 of the trial. The primary outcome alcohol consumption variables were entered together into a MANOVA using Pillai's trace for small samples. These were the percentage of days abstinent, number of heavy drinking days, average drinks per drinking day at week 12. The role of AD status and baclofen (BAC vs. PL) was investigated with “AD,” “baclofen,” and the interaction term “AD × baclofen” in a full factorial model. We placed covariates in the above models that were significantly different following individual ANOVA tests to control for group baseline differences. Due to collinearity between baseline depression and AD use we did not include this baseline characteristic in the model. Frequency of common adverse events associated with baclofen (sedation, skin rash, dizziness) were examined between the non-AD group vs. the AD group among those participants randomized to baclofen using χ^2^-tests.

All analyses were 2-tailed, with significance level at *P* < 0.05. Data were analyzed using SPSS 23 for Mac OSX.

## Results

### Patient baseline characteristics and study variables

Socio demographic and drinking characteristics of this study sample are depicted in Table 2 per treatment group and by AD. ANOVA revealed that baseline demographic and drinking characteristics were not significantly different across baclofen × AD groups (*P* > 0.16) except for DASS anxiety and depression scores (*F* = 3.12, *P* = 0.03; *F* = 3.38, *P* = 0.02, respectively). There were no significant differences between groups on categorical characteristics (χ^2^ < 6.95, *P*'s > 0.07) except for tobacco (χ^2^ < 9.69, *P* < 0.02), depression (χ^2^ < 10.55, *P* = 0.01) and ALD (χ^2^ < 9.69, *P* = 0.05). There were no significant differences in the frequency of dose (25 vs. 75 mg) between AD groups suggesting that the dose of baclofen was distributed evenly among AD groups. There were no significant AD × treatment group differences in study completion rates (*P*'s > 0.72).

**Table 2:** Intention to treat: Baseline characteristics of patients according to concurrent antidepressant use.

**Characteristic**	**Placebo**		**Baclofen 30–75 mg**	
	**AD (*n* = 17)**	**Non-AD (*n* = 16)**	**AD (*n* = 40)**	**Non-AD (*n* = 31)**
Age, y	47.12 ± 10.59	49.00 ± 9.27	48.85 ± 8.99	48.00 ± 11.51
Gender, % F	41	21	32	21
Education, y	14.63 ± 2.55	14.25 ± 3.24	12.66 ± 3.68	13.10 ± 2.86
Unemployed, %	23	50	60	42
Drinks per drinking day^a^	13.81 ± 6.89	14.37 ± 7.23	17.82 ± 12.72	14.74 ± 7.31
Abstinence days before enrolment	2.29 ± 2.23	4.71 ± 7.23	6.27 ± 7.49	3.96 ± 5.52
Years since alcohol-related problems began	13.75 ± 10.48	20.92 ± 12.01	19.18 ± 10.86	16.39 ± 12.25
Cigarette smokers, %^*^	64	64	84	50
Lifetime Major Depression, %+^*^	66	57	81	42
Lifetime Anxiety Disorder, %+	88	70	64	57
ALD, %^*^	29	79	58	57
ADS	17.94 ± 9.18	17.57 ± 9.41	21.63 ± 10.89	17.56 ± 7.96
PACS craving	18.31 ± 6.54	17.36 ± 6.54	17.10 ± 7.95	14.74 ± 7.31
DASS Depression^*^	20.00 ± 13.83	20.29 ± 10.64	18.10 ± 12.15	11.48 ± 7.56

*Data represent mean + SD of raw data unless otherwise noted. There were no significant differences between the groups for continuous variables (P = 0.16) except for DASS Depression (P's < 0.05). There were no differences between groups on categorical variables except for tobacco, ALD and past depression (P's < 0.05)*.

* *P < 0.05*.

a *During the 30 days preceding the first day of the study, based on the Time-Line Follow-Back method*.

*AD, antidepressant; ADS, Alcohol Dependence Severity Scale; PACS, Penn Alcohol Craving Scale; DASS, Depression Anxiety Stress Scale; + as measured by the MINI Neuropsychiatric Diagnostic Interview*.

### Main drinking outcomes

At week 12, drinking data for relapse and lapse was available for 89% of subjects. Table 3 depicts the main outcome measures. Cox regression survival analyses revealed that, for the number of days to first lapse, there was a main effect of baclofen (Log Rank: χ^2^ = 7.47, *P* < 0.05, OR: 2.55, 95%CI: 1.30–4.50) but not for AD (Log Rank: χ^2^ = 0.12, *P* = 0.73, OR: 1.10, 95%CI: 0.62–1.97) and a trend for significance occurred for the baclofen × AD interaction (Log Rank: χ^2^ = 2.98, *P* = 0.08, OR: 0.41, 95%CI: 0.15–1.12). Similarly, for the number of days to relapse, there was a main effect of baclofen (Log Rank: χ^2^ = 7.76, *P* < 0.01, OR: 2.59, 95%CI: 1.33–5.06) but not for AD (Log Rank: χ^2^ = 0.35, *P* = 0.55, OR: 2.00, 95%CI: 0.66–2.17) and a trend for significance occurred for the baclofen × AD interaction (Log Rank: χ^2^ = 3.72, *P* = 0.05, OR: 3.40, 95%CI: 1.01–11.46). MANOVA revealed a significant overall treatment effect attributed to alcohol consumption [Wilks multivariate test of significance; *F*_(3, 62)_ = 3.14, *P* < 0.05] but not for AD [Wilks multivariate test of significance; *F*_(3, 62)_ = 2.10, *P* = 0.11] and a trend for significance occurred for the baclofen × AD interaction [Wilks multivariate test of significance; *F*_(3, 62)_ = 2.51, *P* = 0.07]. Placing significant baseline variables into the models (tobacco, ALD) reduced both the Cox regression and MANOVA AD × baclofen interaction trends (*P*'s > 0.15).

**Table 3:** Intention to treat: drinking outcome measures at week 12 of participants treated with either baclofen (30–75 mg) or placebo according to concurrent antidepressant use.

**Outcome**	**Placebo**		**Baclofen**	
	**AD (*n* = 17)**	**Non-AD (*n* = 16)**	**AD (*n* = 40)**	**Non-AD (*n* = 31)**
**Alcohol consumption measures**				
Time to first lapse (days)± SEM	2.07 ± 0.55	23.08 ± 9.43	29.50 ± 6.13	25.08 ± 6.51
Time to first relapse (days) ± SEM	5.00 ± 2.32	30.23 ± 10.18	35.59 ± 6.37	30.35 ± 6.93
Percentage days abstinent	28.37 ± 28.29	68.10 ± 36.14	68.00 ± 35.26	66.34 ± 28.46
Average drinks per drinking day^+^	7.29 ± 4.49	7.37 ± 9.38	5.46 ± 5.63	5.78 ± 6.91
Number of heavy drinking days^#^^++^	2.83 ± 2.52	1.00 ± 2.21	1.83 ± 2.63	2.18 ± 2.74

*Data represent raw means ± SD unless otherwise noted. Drinks is equal to standard drink (10 g ethanol)*.

# Defined as >4 drinks for women and >5 drinks for men,

+ *at week 12 follow-up*,

++ *per week at week 12 follow-up. AD, antidepressant; SEM, standard error of the mean. There were trends for AD × baclofen interaction effects (P's < 0.08) for time to first lapse (P = 0.08), relapse (P = 0.05) and the combined alcohol consumption variables (P = 0.07: borne out in percentage days abstinent, P = 0.02) although these diminished when controlling for pre-existing baseline differences (ALD, tobacco) (P's > 0.15)*.

### Adverse events

We explored the role of the AD on response to baclofen by comparing the frequency of adverse events between AD among those participants randomized to baclofen (see Table 4). There were no differences between groups for any adverse event (*P*'s > 0.19).

**Table 4:** Side effect profile of subjects treated with either baclofen (30–75 mg) or placebo and concurrent antidepressant use.

**Clinical event**	**Baclofen 30–75 mg**	
	**AD (*n* = 40)**	**Non-AD (*n* = 31)**
	***n* (%)**	***n* (%)**
Sedation or drowsiness	13 (32.50)	10 (32.26)
Dizziness	4 (10.00)	4 (12.90)
Skin rash/itching	5 (12.50)	1 (3.23)
Constipation	3 (7.50)	3 (9.68)
Shortness of breath	2 (5.00)	2 (6.45)
Dry mouth	2 (5.00)	1 (3.23)
Urination problems	1 (2.50)	0 (0.00)
Serious adverse events^*^	4 (10.00)	0 (0.00)

* *P < 0.05, significant difference between AD and non-AD groups randomized to baclofen. AD, antidepressant. In the 75 mg group, 9% (3) of patients reduced the dose due to intolerability (1 patient = 25 mg/day and 2 patients = 50 mg/day). Serious adverse events included one death (not baclofen related), one overdose and two hospitalizations due to intoxication and suicidal ideation*.

## Discussion

The main aim of the current study was to examine the role of concurrent antidepressant use in baclofen treatment response in a randomized, placebo-controlled double blind study. We demonstrated a significant baclofen vs. placebo effect as per previously described (6). We also demonstrated that ADs did not have a significant moderating effect on treatment outcomes at follow-up. We demonstrated a trend for a significant interaction effect of AD (AD vs. non-AD) × baclofen (BAC vs. PL) on days to relapse, days to lapse and alcohol consumption outcomes. Further analysis revealed that this effect is observed in the AD group whereby allocation to placebo resulted in poor outcomes such as shorter time to relapse and lapse. Nonetheless, these trends were diminished when controlling for significantly different baseline characteristics. It is likely that any potential interaction effect is not due to the action of baclofen and ADs *per se* but pre-existing comorbidity in those participants already receiving ADs which lead to shorter relapse in the absence of active treatment.

One retrospective analysis of a clinical trial exploring the relationship between antidepressant use and the alcohol pharmacotherapy naltrexone observed that, for those receiving antidepressants, there was a naltrexone vs. placebo effect on drinking outcomes but this effect was absent for those not receiving antidepressants (19). These results are similar to the trends found in our current results with baclofen (without controlling for pre-exisiting factors) whereby for those receiving anti-depressants, the placebo group displayed shorter times to lapse and relapse that were not seen in those randomized to baclofen. One interpretation is that in these studies patients receiving antidepressants, and presumably with comorbid depression, respond poorly in the absence of active treatment.

We also observed that there were no significant differences on frequency of specific commonly reported adverse events across the AD × baclofen groups. These results should be interpreted with caution given the small sample size, limitations with measuring frequency rather than severity and that the adverse events examined were only those commonly reported across the entire sample. Neuropsychiatric adverse drug reactions such as mania were not systematically examined in this analysis. It is imperative to note that this data was derived from a structured monitored clinical trial with low doses of baclofen whereby patients with an active major mental disorder including bipolar, psychosis or unstable mood, history of suicide attempt or recent commencement of ADs were excluded from the trial. Indeed, there have been case reports of baclofen-induced manic symptoms in the literature yet these have generally occurred at higher doses of baclofen during the dose-increase phase (e.g., up to 180 mg/d) but mainly in patients with a history of bipolar disorder (20).

There are several limitations of the current study. Given the trend for significance of interaction effects it is possible that we had limited power to detect a significant effect. In addition, the study is a retrospective analysis and was not designed to examine ADs and baclofen such that baseline characteristics were not balanced. However, we did control for these in our analyses which suggested that the trends for interaction effects were due to pre-existing factors. Longitudinal modeling of symptoms of depression comparing AD and non-AD groups during baclofen treatment would further elucidate this relationship. Further, it would be important to determine any interaction effect of the two medications including mood and side effects in participants that commence ADs in conjunction or within a similar time frame to baclofen. Indeed, participants receiving ADs in the current study were required to be stable and to have commenced more than 2 months prior to baclofen. Participants in the current study were provided a thorough assessment of mood stability before enrolment and monitored for safety throughout as part of the clinical trial schedule. It is possible that this level of monitoring may not be provided in the wider community which may limit the generalizability of these results. Finally, while we have some diagnostic information regarding mood and anxiety disorders in this sample, we do not have direct evidence regarding the clinical indication for why the antidepressants were initially prescribed.

## Conclusion

This is the first study to investigate the moderating role of anti-depressant use on response to baclofen. Our results suggest that the concurrent receipt of antidepressants commenced more than 2 months prior to baclofen treatment in less complex alcohol dependent patients (with no history of bipolar or suicide attempt) does not negatively impact on drinking outcomes. Trends for an interaction effect between antidepressants and baclofen that were observed for all drinking outcomes may be due to pre-existing factors in the antidepressant group which lead to poor outcomes in the absence of active treatment (i.e., placebo allocation). Well-controlled prospective clinical research examining the effect of commencing antidepressants on neuropsychiatric adverse events during baclofen treatment is required.

## Author contributions

KM supervised the study, analysis of data, and writing of the manuscript. SH wrote the manuscript and completed data presentation and analysis. NJ assisted in writing of the manuscript and interpretation. AB assisted supervision of the study, interpretation and writing of the manuscript. EL completed the psychological assessments and assisted writing the manuscript. NP and GD were site physicians. PH supervised the overall conduct of the study and was the lead physician.

### Conflict of interest statement

The authors declare that the research was conducted in the absence of any commercial or financial relationships that could be construed as a potential conflict of interest.
